# Carrier-Free Microspheres of an Anti-Cancer Drug Synthesized via a Sodium Catalyst for Controlled-Release Drug Delivery

**DOI:** 10.3390/ma11020281

**Published:** 2018-02-11

**Authors:** Yong Xie, Xinxin Ma, Xujie Liu, Qingming Long, Yu Wang, Youwei Yao, Qiang Cai

**Affiliations:** 1 Graduate School at Shenzhen, Tsinghua University, Shenzhen 518055, China; jy15@mails.tsinghua.edu.cn (Y.X.); 13303275396@163.com (Q.L.); yaoyw@sz.tsinghua.edu.cn (Y.Y.); 2 State Key Laboratory of New Ceramics and Fine Processing, School of Materials Science and Engineering, Tsinghua University, Beijing 100084, China; zpheadwang@163.com; 3 Southern China Branch, Sinopec Commercial Holding Company Limited, Guangzhou 510630, China; 15201106417@163.com

**Keywords:** carrier-free, 4-amino-2-benzyl-6-methylpyrimidine, biomaterial, drug delivery

## Abstract

There are several challenges involved in the development of effective anti-cancer drugs, including accurate drug delivery without toxic side effects. Possible systemic toxicity and the rapid biodegradation of drug carriers are potential risks in the use of carriers for drug-delivery formulations. Therefore, the carrier-free drug delivery of an anti-cancer drug is desirable. Herein, 4-amino-2-benzyl-6-methylpyrimidine (ABMP) was synthesized via a new method using a sodium catalyst, and proved to be effective in inducing breast cancer cell (MDA-MB-231) apoptosis. Moreover, the transparent amorphous state solid of ABMP was demonstrated to have a slow-release property in phosphate buffer solution (PBS). Microspheres of ABMP were prepared with diameters in the range of 5–15 μm. The slow-release property of the ABMP microspheres indicated their potential use for controlled-release drug delivery. We believe that microspheres of ABMP have potential as a new kind of carrier-free anti-cancer drug delivery system.

## 1. Introduction

Breast cancer has become one of the most prevalent diseases, and is a leading cause of death worldwide [1,2,3,4,5]. Currently, considerable research is being carried out toward the development of anti-cancer drugs [6,7,8]. Some anti-cancer drugs, including paclitaxel and doxorubicin, have proved to be powerful in inducing apoptosis [9,10,11,12]. However, there are still many difficulties that hinder the development of chemotherapies using anti-cancer drugs. First, most anti-cancer drugs are extracted from existing organisms, and the artificial synthesis is often difficult [13,14]. In addition, the development of controlled-release formulations of anti-cancer drugs presents several challenges, including the low water solubility of many anti-cancer drugs, and the development of drug carriers without systemic toxicity and rapid biodegradation. Considering the problems mentioned above, a new drug delivery formulation is desirable.

It is well known that the controlled release of anti-cancer drugs plays an important role in cancer therapy. In response to problems that have occurred with the controlled release of anti-cancer drugs, considerable research has been performed, and some formulations have proved to be effective in model systems [15,16,17]. Drug carriers that have been designed and modified for use with anti-cancer drugs include mesoporous/microporous particles, vesicles, multifunctional dendritic polymers, micelles, and nanocapsules, and some of these formulations can also provide targeted release [18,19,20,21,22,23]. However, despite the merits of these drug carriers, many problems remain. The drug-loading capacity is often low, because the carriers are usually the major component, with a weight far greater than the drugs (thus, loading is generally less than 10%) [24]. Other problems include the possible systemic toxicity and biodegradation of the carriers [25]. The drug carriers are designed to deliver a drug to a diseased region with the expectation that the carriers are inert and nontoxic. However, the systemic toxicity and subsequent degradation of carriers causes potential problems, including imposing an extra burden on the patients, and the mechanisms by which this occurs are still unclear. As a result, efforts have been made toward developing a carrier-free drug delivery system. In general, some kind of polymer and functionalized organic matter are introduced to prepare a carrier-free drug delivery system [26,27]. However, it is not known whether these introduced materials will bring new risks. There are some other methods for the preparation of carrier-free drug delivery systems, including the micronization or nanonization of solid anti-cancer drugs [28,29], but the crystallization of the drug particles is a problem with this method, which limits its application [30].

Therefore, the synthesis of anti-cancer drugs that possess certain physicochemical properties is desirable for carrier-free drug delivery [31]. An anti-cancer drug that has the property of consolidation forming after quenching is an ideal material for designing carrier-free drug delivery formulations. Amino pyrimidine is a common core structure in anti-cancer drugs, and a series of amino pyrimidines have been studied and shown to be potential anti-cancer drugs [32,33,34], including 4-amino-2-benzyl-6-methylpyrimidine [35]. The traditional route for amino pyrimidine synthesis using guanidine and imine involves complicated procedures and high costs. The oligomerization of nitriles catalyzed by a base and an organic salt has been reported for the synthesis of the amino pyrimidine structure [36,37,38]. Sodium methoxide, IrH(CO)(PPh_3_)_3_, and sodium hydride have been employed as catalysts in this synthesis [39,40,41,42,43]. Considering the potential application of ABMP as an anti-cancer drug, a clean, simple, low-cost, environment-friendly, and effective method is desired for the synthesis of ABMP.

The establishment of an anti-cancer drug delivery model containing a carrier-free drug is highly desirable. In order to achieve carrier-free anti-cancer drug microspheres for a potential drug delivery system, a new strategy is proposed in this work. First, a new method for the synthesis of 4-amino-2-benzyl-6-methylpyrimidine (logogram: ABMP) is demonstrated. Meanwhile, the ABMP was proved to be effective in inducing human breast cancer cell (MDA-MB-231) apoptosis. This was followed by the preparation of microspheres of ABMP in a high-temperature and high-pressure system. Release tests of the transparent amorphous state solid cube and the microspheres of ABMP were also performed.

## 2. Materials and Methods

### 2.1. Preparation of ABMP

The experiment was carried out in an anaerobic glove box to provide water-free and oxygen-free conditions. Benzyl cyanide (500 mL) was put into a 1000-mL balloon flask, and then heated to 80 °C in an oil bath. Afterwards, small pieces of sodium were added into the benzyl cyanide, piece by piece, with continuous stirring. After 12 h, the reaction solution turned into a brown solid, then recrystallization was employed to purify the product to give ABMP as a light pink solid in approximately 90% yield.

### 2.2. Characterization

The solubility of the synthesized 4-amino-2-benzyl-6-methylpyrimidine in various solvents, including ethanol (EtOH), water (H_2_O), dimethyl sulfoxide (DMSO), and dimethylformamide (DMF), was investigated at a concentration of 5% (*w*/*v*). The Fourier transform infrared (FT-IR) spectrum of the synthesized amino pyrimidine was measured in the range of 4000–400 cm^−1^ on a FT-IR spectrometer (6700FTIR, Thermo Fisher Scientific, Waltham, MA, USA). ^1^H and ^13^C nuclear magnetic resonance (^1^H-NMR and ^13^C-NMR) spectra were obtained in DMSO-*d*_6_ using a 600-MHz NMR spectrometer (JNM-ECA600, JEOL, Akishima, Japan). Single crystal X-ray diffraction (SXRD) was executed in a single-crystal diffractometer (RIGAKU, R-Axis-Spider, Tokyo, Japan). The liquid chromatograph-mass spectra (LC-MS) of the synthesized amino pyrimidine was analyzed in a liquid chromatography-mass spectrometer (LTQ, Thermo Fisher Scientific, Waltham, MA, USA). The three-dimensional (3D) photoluminescence spectra of the synthesized amino pyrimidine were obtained using a fluorescence spectrometer (FLSP920, Edinburgh Instruments Ltd., Livingston, U.K.) with the concentration of samples set to 1% (*w*/*v*). The thermogravimetric-differential scanning calorimetry (TG-DSC) analysis was acquired from a simultaneous thermal analyzer (STA449F3, NETZSCH-Gerätebau GmbH, Selb, Germany).

### 2.3. Cell Culture

The human mammary carcinoma cell line (MDA-MB-231), the immortalized cell line of mouse fibroblast cells (L929), and the autologous bone marrow stem cell (BMSC) were selected as the experimental cell line to evaluate the effect of the synthesized 4-amino-2-benzyl-6-methylpyrimidine in inducing cancer cell apoptosis. The MDA-MB-231 cells were cultivated in L-15 (Invitrogen, Carlsbad, CA, USA) medium with 15% fetal bovine serum (FBS; Invitrogen, USA), 100 μg/mL streptomycin, and 100 μg/mL penicillin under standard culture conditions (5% CO_2_, 37 °C). The cells (purchased from the Chinese Academy of Medical Sciences, Beijing, China) were expanded, and the medium was replaced with fresh medium every two days. Different concentrations, i.e., 0, 10, 25, 50, 75, 100, 125, and 150 μg/mL, of synthesized 4-amino-2-benzyl-6-methylpyrimidine were used to treat the cells.

### 2.4. CCK-8 Assays

The CCK-8 assay was used to observe the number of living cells. A 48-well plate was used to seed the cells at a density of 15,000 cells per well. After seeding for 24 and 48 h within in vitro assays, the CCK-8 assay was performed. Briefly, CCK-8 (Dojindo, Kyushu, Japan) was added to each well with a 10 vol % of the medium. The absorbance (OD) of the solution, after incubation for 2 h at 37 °C, was measured by a microplate reader at 450 nm. The experiments were carried out in quadruplicate.

### 2.5. Cell Cycle Analysis

MDA-MB-231 cells were plated at a density of 1 × 10^6^ cells/well on six-well plates. After treatment with ABMP, both detached and attached cells were collected into flow cytometry tubes, and centrifuged at 1000 rpm for 5 min to obtain a cell pellet. The cells were suspended in a solution of phosphate-buffered saline (PBS)/ ethylene diamine tetraacetic acid (EDTA), and an equal volume of ethanol was added to the cells, which were then incubated for 30 min at room temperature. Cells were collected by centrifugation. For cell cycle analysis, the ethanol-fixed cells were stained with propidium iodide (PI) in the presence of RNase A, and then analyzed by a fluorescence-activated cell sorter (FACS). In each analysis, 30,000 events were recorded.

### 2.6. Apoptosis Assay

For analyzing apoptosis, fluorescein isothiocyanate (FITC)-conjugated annexin V binding and PI staining were performed using a kit from Multi Sciences. MDA-MB-231 cells were plated at a density of l × 10^6^ cells/well on six-well plates, and cell apoptosis was induced by ABMP treatment for 24 h. Both detached and attached cells were collected for FITC-conjugated annexin V and PI staining. Early and late apoptotic cell populations were visualized by constructing a dot plot with the aid of FACS. The FL1 channel was used to detect annexin V-FITC staining, and the FL2 channel was used for PI staining.

### 2.7. Exploration of Amorphous State Solidification

ABMP was formed as a transparent amorphous state solid via a quenching process. Synthesized ABMP (1 g) was put into a 20-mL glass bottle and heated to 120 °C in an oil bath for 10 min until a liquid phase was obtained. Then, the liquid was poured into a glass cube mold, which was placed in a cold environment. After a few minutes, a transparent amorphous state solid was formed. Repeated stretch and compression operations were executed, and soft stripes were recorded by a camera.

### 2.8. Slow-Release of Ass-ABMP

The method for drug release in a PBS solution, as reported previously, was applied to explore the potential for the carrier-free release of the amorphous state solid of ABMP. A standard curve was acquired on a UV-VIS spectrophotometer (Agilent Technologies Inc., Santa Clara, CA, USA) before the release test. Then, a cube of amorphous state solid ABMP was put into 50 mL of PBS solution with stirring. Dissolution medium (2 mL) was replaced by fresh PBS at predetermined time intervals for determination of the drug concentration.

### 2.9. Preparation of Spheroidal Particles

Spherical particles were acquired by the following procedure. Briefly, a homogeneous mixed solution containing 10 mL of deionized water and 1.5 g of Tween-40 was prepared and transferred to a 30-mL Teflon vessel. Then, 0.2 g of synthesized 4-amino-2-benzyl-6-methylpyrimidine (ABMP) was added. The mixture was heated to 150 °C in an oven, and then vigorous shaking was adopted, before a cool environment was provided for quick cooling. Then, 200 μL of product was placed on a piece of microslide, and the morphology was observed under an optical microscope.

### 2.10. Carrier-Free Release of Spheroidal Particles

A solution of ABMP microspheres (3 mL) was put into 50 mL of fresh phosphate-buffered saline (PBS) with stirring at 37 °C. A 0.2-mL aliquot of solution was replaced by fresh PBS after 10, 20, and 40 h. The morphology of the microspheres was observed by an optical microscope.

### 2.11. Statistical Analysis

The results were expressed as mean ± standard deviation (SD). The statistical significances of differences in means were determined by the two-tailed Student *t*-test, or one-way or two-way analysis of variance (ANOVA), followed by post hoc comparisons with the least significant difference (LSD) method using SPSS 19.0 software (19th Edition, International Business Machines Corporation, Armonk, NY, USA). A value of *p* < 0.05 was considered as statistically significant.

## 3. Results and Discussion

### 3.1. Oligomerization of Acetonitrile

**Figure 1:**
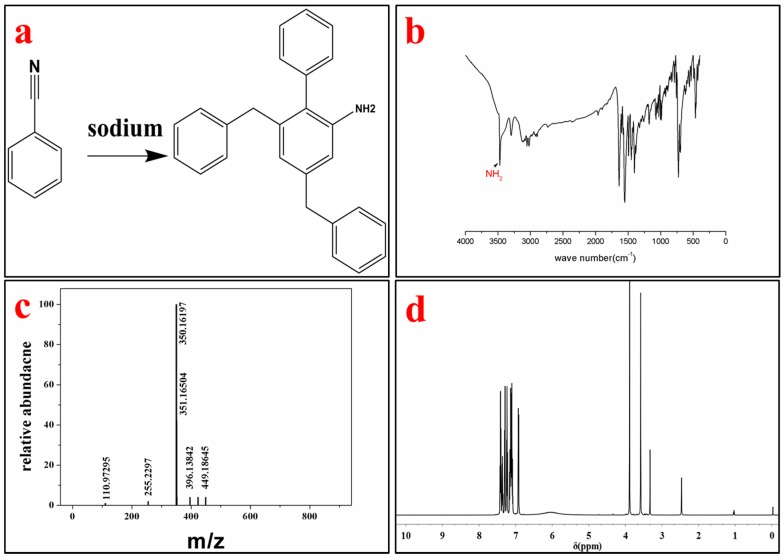
Synthesis and characterization of 4-Amino-2-benzyl-6-methylpyrimidine (ABMP). (**a**) Chemical structure of ABMP synthesized from benzyl cyanide using sodium as a catalyst; (**b**) Fourier transform infrared (FT-IR) spectrum of ABMP; (**c**) mass spectrum of ABMP; and (**d**) ^1^H NMR spectrum of ABMP.

**Figure 2:**
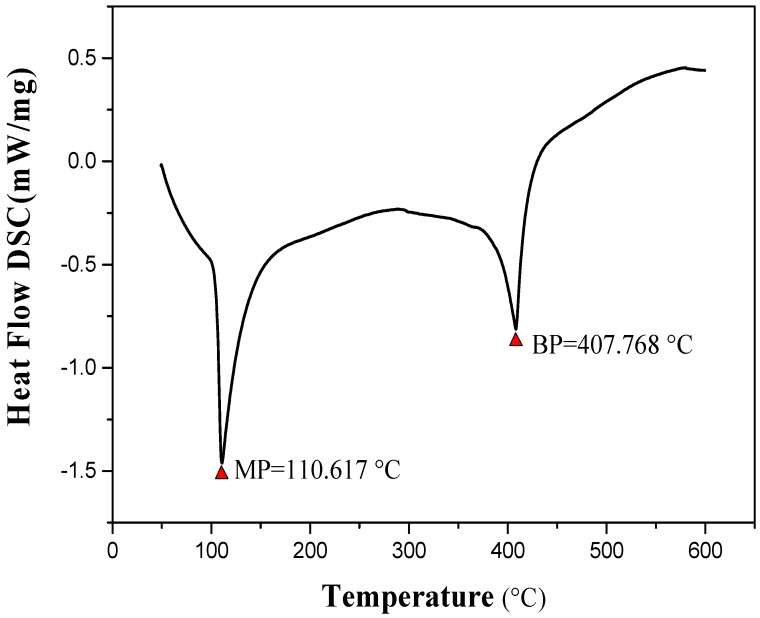
Differential thermal analysis of ABMP; the melting point is 110.617 °C, and the boiling point is 407.768 °C.

The product was identified by different characterization methods, including ^1^H and ^13^C NMR, FT-IR, LC-MS and SXRD. The lattice constants of the ABMP cell are listed in Table 1. The results indicated that the ABMP cell belongs to the triclinic crystal system. The FT-IR and LC-MS spectra of the obtained product are given in Figure 1b,c, respectively. As shown in Figure 1b, the IR bands at approximately 3475 and 3296 cm^−1^ are dominated by the N–H stretch vibration contribution, the 2903 and 1480 cm^−1^ bands indicate the existence of CH_2_, and the bands at 3030 and 1600–1500 cm^−1^ are attributed to the vibrations of aromatic nuclei. The LC-MS spectrum not only verifies the molecular weight, it also gives an indication of the purity of the product. ^1^H NMR (600 MHz, DMSO-*d_6_*, δ/ppm): 7.51–7.04 (m, 15H, br), 6.92 (d, 2H, NH_2_), 3.88 (s, 4H, CH_2_) (Figure 1d). The melting point of ABMP is 110 °C, and the boiling point is 408 °C, which was confirmed by TG-DSC analysis, as shown in Figure 2.

It is difficult to polymerize or oligomerize the carbon-nitrogen triple bonds in acetonitrile unless severe conditions are imposed. In our previous study [44], an oligomer of acetonitrile with polymerization degree *n* = 17 was synthesized via an electrochemical method. In addition, a cyclotrime of acetonitrile was acquired using sodium as a catalyst under ambient conditions. The cyclotrime of acetonitrile had the ability to induce osteogenesis [45]. The α-proton found in nitriles makes it possible for the α-carbon to be changed into a carbanion, especially when sodium is employed. The super catalyst ability of the carbanion has the essential effect in the formation of amino pyrimidine. As is mentioned above, the one-pot method for amino pyrimidine synthesis using sodium as a catalyst has been proved to be versatile for the synthesis of small nitriles, such as acetonitrile and benzyl cyanide. There are other sites exposed, which can be used to graft various groups aimed at targeted therapy.

### 3.2. Fluorescent Properties

**Figure 3:**
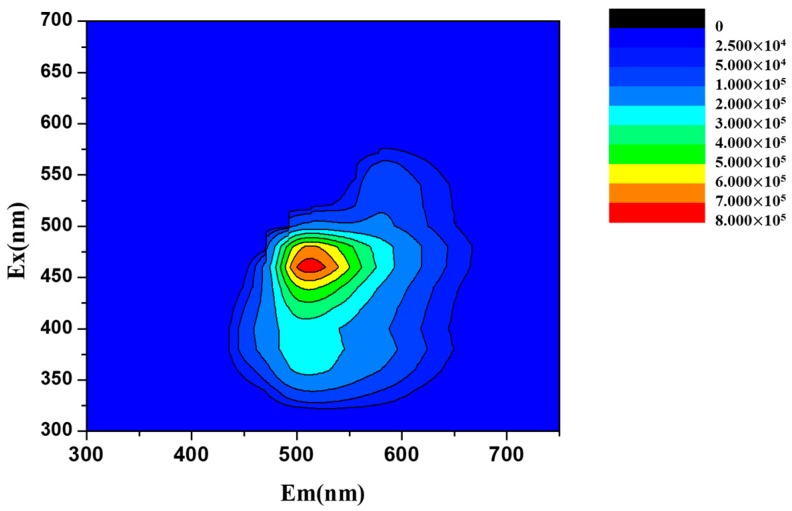
Three-dimensional (3D) photoluminescence spectra of ABMP in ethanol with the concentration of the sample set to 1% (*w*/*v*).

Encouraged by the integration of diagnostics and therapeutics, the fluorescent properties of drug candidates have become of interest in drug design and synthesis. The 3D photoluminescence spectra (Figure 3) of ABMP was acquired on a fluorescence spectrometer with the concentration of the sample set to 1% (*w*/*v*). The maximum fluorescent intensity occurs at 465 nm/525 nm (excitation/emission). The conjugated π–π structure provides a theoretical foundation for the fluorescent properties. In addition, the solubility of ABMP was measured at the same time, and the results indicated that it is slightly soluble in deionized water, and very soluble in ethanol (EtOH), dimethyl sulfoxide (DMSO), dimethylformamide (DMF), and tetrahydrofuran.

### 3.3. Ability to Induce Cancer Cell Apoptosis

#### 3.3.1. ABMP Inhibited MDA-MB-231 Breast Cancer Cell Proliferation

**Figure 4:**
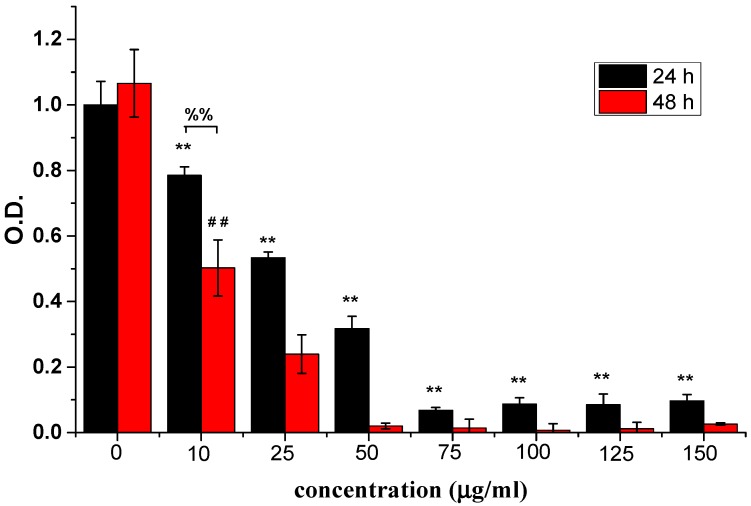
Relative cell numbers determined by the absorbance (OD) value using CCK-8 at 24 and 48 h. Breast cancer cells (MDA-MB-231) were treated with ABMP at different concentrations, i.e., 0, 10, 25, 50, 75, 100, 125, and 150 μg/mL. The experiments were carried out in quadruplicate. Double asterisks (**) and double pounds (##) refer to statistical significance *p* < 0.01 compared with control groups. Double percent (%%) refers to a statistical significance of *p* < 0.01 between the OD values after seeding for 24 and 48 h.

**Figure 5:**
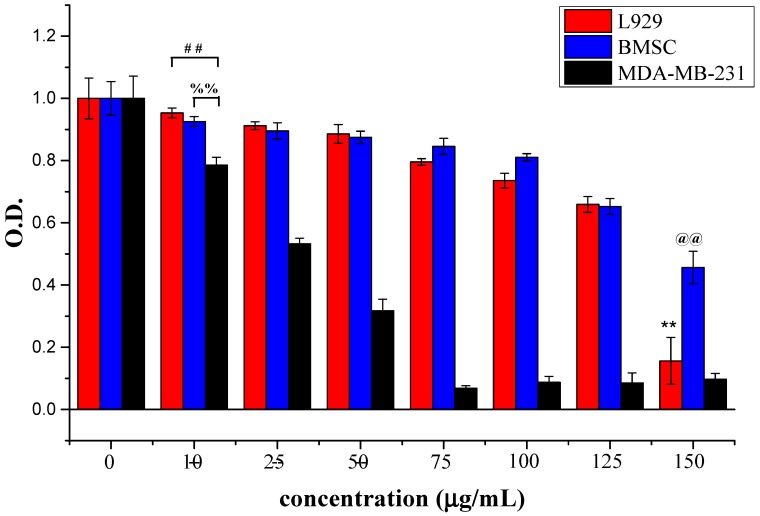
Relative cell numbers determined by the OD value using CCK-8 at 24 h. L929, bone marrow stem cell (BMSC), and MDA-MB-231 cells were treated with ABMP at different concentrations, i.e., 0, 10, 25, 50, 75, 100, 125, and 150 μg/mL. The experiments were carried out in quadruplicate. Double asterisks (**) and double at (@@) refer to statistical significance *p* < 0.01 compared with control groups. Double pound (##) refers to a statistical significance of *p* < 0.01 between the OD values of L929 and MDA-MB-231 after seeding for 24 h; double percent (%%) refers to a statistical significance of *p* < 0.01 between the OD values of BMSC and MDA-MB-231 after seeding for 24 h.

**Figure 6:**
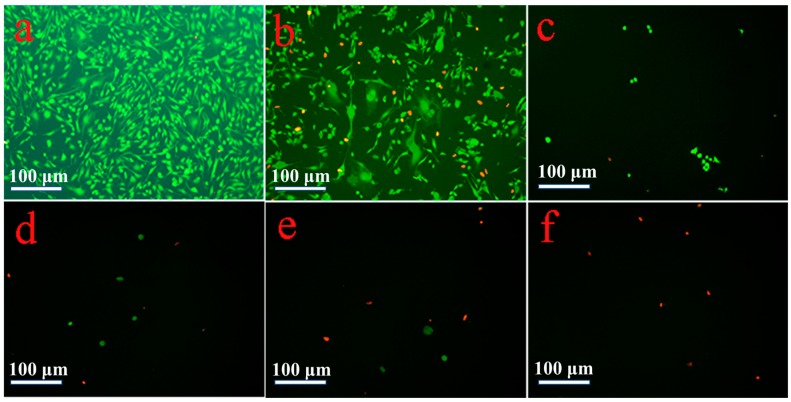
Cell numbers and viability evaluated using calcein-AM/ propidium iodide (PI) staining after 48-h seeding. (**a**) Control; (**b**) 25 μg/mL; (**c**) 50 μg/mL; (**d**) 75 μg/mL; (**e**) 100 μg/mL; and (**f**) 125 μg/mL.

The inhibitory effects of ABMP on human breast cancer cells was assessed using MDA-MB-231 cells. MDA-MB-231 cells are a type of human breast cancer cell that are widely used to evaluate the efficacy of anti-cancer drugs [46]. Different concentrations, i.e., 0, 10, 25, 50, 75, 100, 125, and 150 μg/mL of ABMP were used to treat MDA-MB-231 cells. The relative cell numbers determined by the OD value using CCK-8 at days 1 and 2 are shown in Figure 4. The results suggested that ABMP inhibited MDA-MB-231 breast cancer cell proliferation in a dose and time-dependent manner. After 24-h treatment with 25 μg/mL ABMP, the percentage of dead cells was ~50%, and by 48 h, this had increased to 79%. At a dosage of 50 μg/mL of ABMP, the percentage of dead cells increased to 69% after 24 h, and 99% at 48 h. The IC_50_ value of MDA-MB-231 for ABMP was 25 μg/mL (71 nM). Meanwhile, the contrastive experiment was executed, and the results were shown in Figure 5. It turned out that the normal cell viability of L929 and BMSC was slightly influenced (the number of cell deaths is less than 80%) after treatment with ABMP at concentrations between 10–75 μg/mL, suggesting a low cytotoxicity in this concentration range. Furthermore, the MDA-MB-231 cell numbers and viability were evaluated using calcein-AM (3′,6′-Di(*O*-acetyl)-4′,5′-bis[*N*,*N*-bis(carboxymethyl)aminomethyl]fluorescein, tetraacetoxymethyl ester)/PI (phosphatidylinositol) staining after 48-h seeding, and the results (Figure 6) were collected after 48-h seeding. In Figure 6, the cells with green are alive, while dead cells are shown in red. The cell numbers and viability were observed to gradually decrease with an increase in ABMP concentration. When 150 μg/mL of ABMP was employed for 48 h, MDA-MB-231 cells were almost completely annihilated, which demonstrated a good performance of ABMP in inhibiting cancer cell proliferation.

#### 3.3.2. ABMP-Induced Apoptosis of MDA-MB-321 Cancer Cells

**Figure 7:**
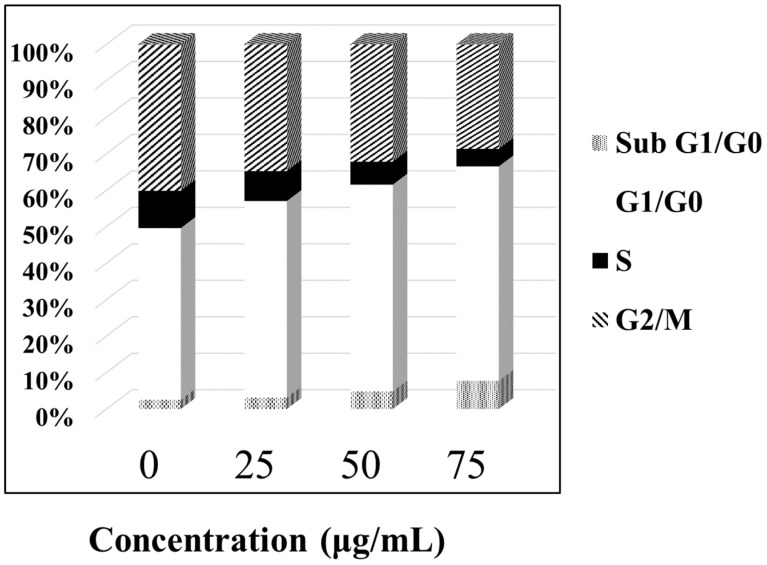
ABMP arrests MDA-MB-231 cells in G0/G1. Proliferating MDA-MB-231 cells were treated with various concentrations of ABMP for 24 h, fixed with ethanol, and stained with PI. Cell cycle distributions were analyzed by fluorescence-activated cell sorter (FACS) analysis.

**Figure 8:**
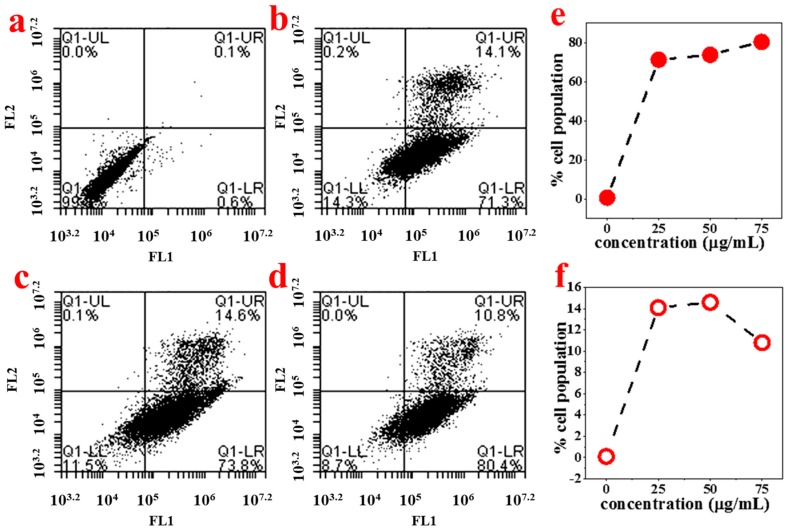
Histograms from FACS analysis at each ABMP concentration. The FL1 channel was used to detect annexin V-FITC staining, and the FL2 channel was for PI staining. (**a**) Control; (**b**) 25 μg/mL; (**c**) 50 μg/mL; (**d**) 75 μg/mL; (**e**) percentage of early apoptotic cells stained with annexin-V-FITC at various concentrations of ABMP; (**f**) percentage of late apoptotic or necrotic cells stained with both annexin V-FITC and PI at various concentrations of ABMP.

In order to study the mechanism of antiproliferative activity by ABMP, the cell cycle distributions of MDA-MB-231 cells affected by ABMP were analyzed in detail. Cells were treated with various concentrations of ABMP for 24 h and subject to FACS analysis after PI staining of the chromosomal DNA. In histograms of FACS analysis, untreated proliferative MDA-MB-231 cells showed cell cycle distributions of 47.12% in G1/G0, 10.18% in S, 40.24% in G2/M, and 2.46% in the sub-G1/G0 phase. However, after ABMP treatment, G1/G0 and sub G1/GO populations increased in an ABMP-dose dependent manner. At 75 μg/mL of ABMP, the populations reached a minimum of 4.73% for S, and 28.72% for G2/M. In contrast, the populations increased up to 58.82% in the G1/G0 phase, and 7.73% in the sub-G1/G0 phase after treatment with 75 μg/mL of ABMP (Figure 7). These data indicated that ABMP has an activity to arrest MDA-MB-231 cell growth in G0/G1. The simultaneous staining of cells with annexin-V and PI dye made it possible to distinguish between early apoptotic cells (stained positive for annexin-V and negative for PI), and late apoptotic or necrotic cells (stained positive for both annexin-V and PI); the results were shown in Figure 8. In MDA-MB-231 control culture, the number of apoptotic cells is negligible (Figure 8a). After 24 h of treatment with ABMP, the population in the early stages continuously increased with the amount of treated ABMP, giving a maximum value of about 80.4% at 75 μg/mL of ABMP (Figure 8d). On the other hand, the population of cancer cells in the late apoptotic or necrotic stage significantly increased. It reached a maximum value of approximately 14.6% at 50 μg/mL of ABMP, then slightly decreased from this maximum value as the concentration of ABMP became higher. These results indicated that the persistent presence of ABMP at concentrations of 25–75 μg/mL can cause significant MDA-MB-231 cell death through the apoptotic pathways.

Paclitaxel and doxorubicin are known to have an effect on inducing cancer cell apoptosis and have been in clinical use, but there are still many existing limitations that hinder their clinical application, including the high cost caused by the complicated procedures for preparation, poor aqueous solubility, and the need for a carrier for controlled release. Although ABMP has a high IC_50_ value for MDA-MB-231 cells (71.22 nM) compared with paclitaxel (2.4 nM) [47], there are some advantages for ABMP over traditional anti-cancer drugs. First, the method for the preparation of ABMP has been proved to be useful for the synthesis of drugs containing pyrimidine via a bottom-up process, which provided more opportunities to construct new molecules with desired biocompatibility. Second, the ABMP system is solvent-free, simple, inexpensive, environmentally friendly, and has fluorescent properties, which gives the ABMP system advantages over traditional drugs, despite a reduction in the induction of cell apoptosis. The toxicity in vivo, and the mechanism by which ABMP induces cancer cell apoptosis, will be explored in future work by our group.

### 3.4. Potential of ABMP as a Biomaterial in a Carrier-Free Release Drug Model

**Figure 9:**
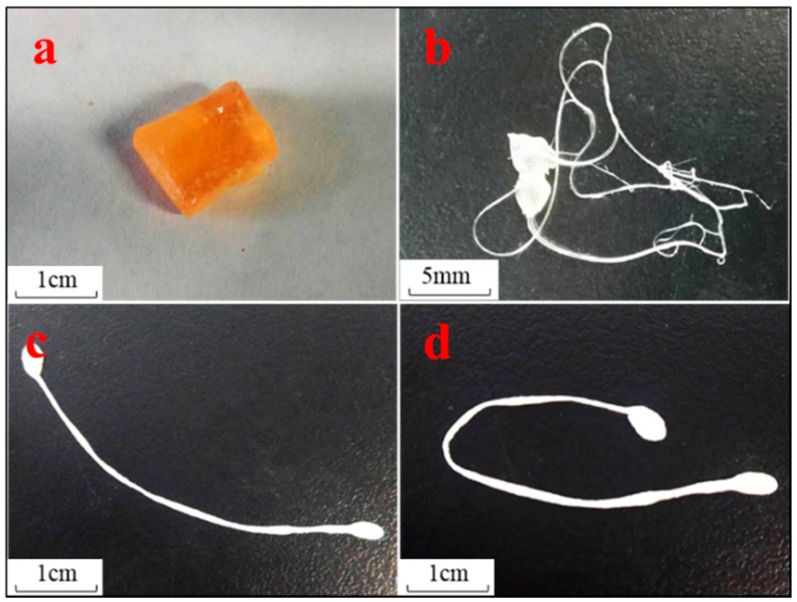
Transparent amorphous state solid of ABMP. (**a**) Appearance of amorphous state solid ABMP (ass-ABMP); (**b**) filiform appearance of ass-ABMP when an outside force was employed; (**c**,**d**) deformation is still possible after stretching several times.

**Figure 10:**
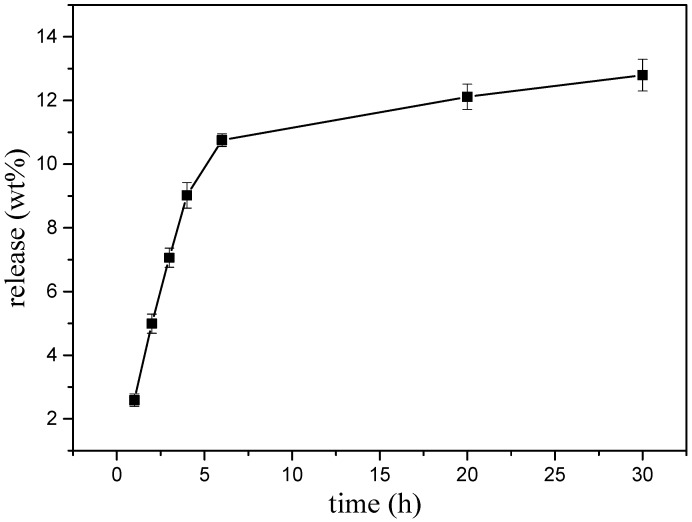
Slow-release curve of ass-ABMP in phosphate-buffered saline (PBS) solution. The test was executed three times under the same conditions.

A preliminary experiment was executed to explore the possibility that ABMP may form a solid state with a slow-release ability. A transparent amorphous state solid was formed when ABMP was heated to 120 °C and rapidly cooled (Figure 9a). The rapid cooling made it difficult for the unordered molecules in the liquid to form crystals so that the mixing and enwinding of side groups occurred, and thus, an amorphous state was formed. The transparency was controlled by the condensate depression. Moreover, the transparent amorphous state solid ABMP (ass-ABMP) was transformable when an outside force was employed. Ass-ABMP could be stretched like polymer materials (Figure 9b), and the performance could be easily repeated. (Figure 9c,d). In addition, ass-ABMP had a slow dissolution ability in water and ethanol. An elaborate test was executed to explore whether ass-ABMP has the potential for slow release (Figure 10). A cube of ass-ABMP was added to 100 mL of PBS solution with continuous stirring. In the first few hours, the ass-ABMP showed fast-release behavior, because of the high concentration difference. When the saturation concentration occurred after 10 h, the release speed became quite sluggish. It was observed that only 12.79% was released after 30 h, and the release curve was in line with those for particles used for drug delivery; this provided strong evidence that ass-ABMP has the potential for slow release. The ^1^H NMR spectra demonstrated that the solidification was a physical change only, and the structure of ABMP was not changed after dissolution in either water or PBS.

### 3.5. Microspheres of ABMP

**Figure 11:**
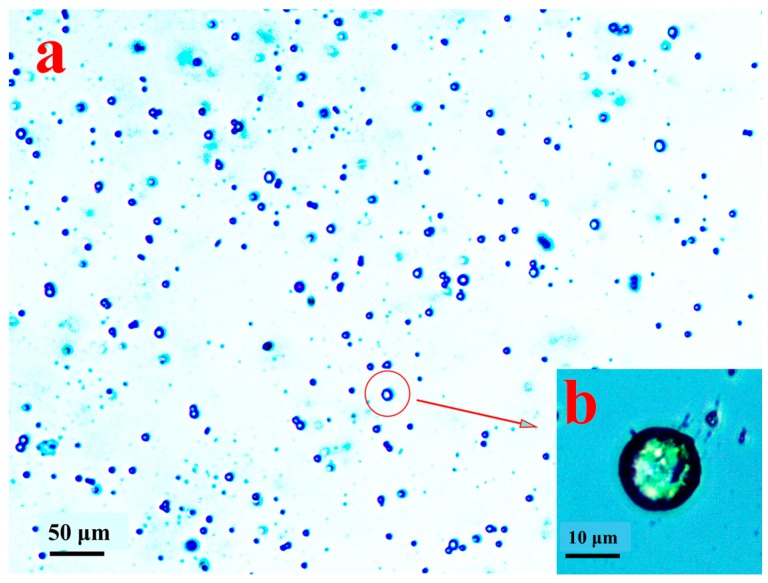
Morphology of microspheres of ABMP under an optical microscope: (**a**) overall distribution in a low-power image (100 times); and (**b**) the detailed morphology in a high-power image (400 times).

The morphology of the prepared microspheres of ABMP is shown in Figure 11. The diameter of the microspheres was in the range 5–15 μm, and the microspheres were separate to each other (Figure 11a). When the preparation was heated to 150 °C, ABMP became liquid, which was mixed with water. Then, when quenched with violent shaking, the tween-40 packed the ABMP into a sphere, and the liquid phase solidified from the quenching, and turned into the amorphous state. The detailed morphology is displayed in Figure 11b, in which the size and the solidification state of the ABMP microspheres can be seen.

**Figure 12:**
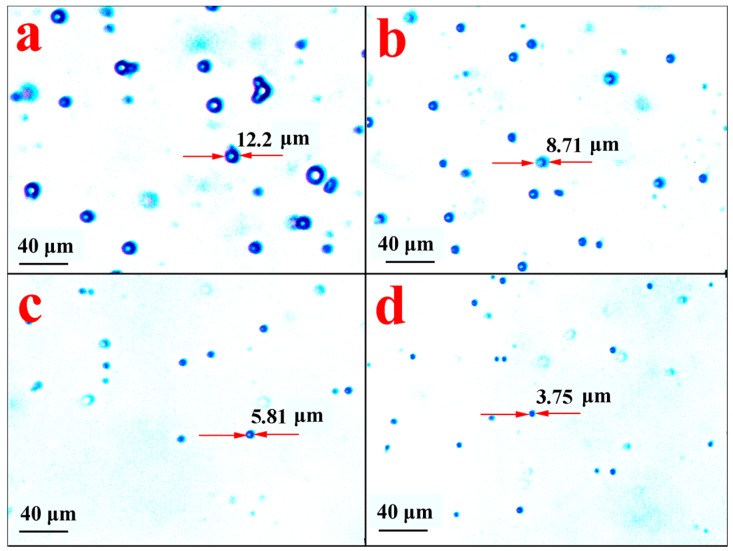
Morphology of ABMP microspheres under an optical microscope after release for (**a**) 0 h; (**b**) 10 h; (**c**) 20 h; and (**d**) 40 h in PBS. The diameter of a typical particle is labeled in each picture.

Using PBS as a simulative fluid condition at 37 °C, the slow-release behavior of ABMP microspheres was assessed by observing the morphological changes of the microspheres. The microspheres were investigated at different time points, and the observed morphology is shown in Figure 12. Figure 12a shows the morphology of the control group, and Figure 12b–d show the morphology of the microsphere after 10, 20, and 40 h, respectively. The size of the microspheres became smaller with an increase in the release time. The release proportion was assessed according to the loss in the volume of the microsphere. The results indicated that 63.7%, 89.2%, and 97.2% of ABMP was released into the PBS after 10, 20, and 40 h, respectively. Compared with the ass-ABMP cube, the dissolution velocity was high.

Microspheres that consisted of only an anti-cancer drug have been prepared and proved to be effective in the slow release of ABMP. These results provide a new research strategy for the design of carrier-free drug release formulations. Unlike other methods for preparing carrier-free drug delivery systems, such as the solvent exchange [48] or micronization/nanonization methods [49], the employed emulgator-assisted solidification method in this study resulted in naked and amorphous microspheres of an anti-cancer drug. In addition, efforts to decrease the size of the microspheres are underway in our group. Meanwhile, more detailed studies investigating the anti-cancer mechanism and the anti-cancer effect in vivo are also being pursued.

## 4. Conclusions

A new sodium catalytic reaction applied to benzyl cyanide cyclopolymerization to synthesize amino pyrimidine has been developed. The method is solvent-free, simple, inexpensive, and environment-friendly, which increases its potential for application in the design of medicinal molecules. The developed method using sodium as a catalyst can be also applied to the synthesis of other amino pyrimidine structures with different side chains, using different small nitrile molecules, which may also have potential in biomedical applications. Moreover, ABMP, the main product of the benzyl cyanide cyclopolymerization reaction, possesses many gratifying characteristics for a potential anti-cancer drug. In particular, it has a satisfactory IC_50_ value (25 μg/mL), combined with intrinsic fluorescent properties.

In addition, as a transparent amorphous state solid, ABMP shows carrier-free release behavior, which may be used in drug delivery systems. The ass-ABMP release test was the first attempt at using an amorphous state solid amino pyrimidine anti-cancer drug for carrier-free drug release. In addition, microspheres of ABMP were prepared by a new method. A release test indicated that the microspheres of ABMP have the potential for controlled release in a carrier-free drug delivery model.

## Figures and Tables

**Table 1:** Lattice constants of the ABMP cell measured by single crystal X-ray diffraction (SXRD).

a	b	c	α	β	γ	Crystal System
15.1476	17.3671	23.1182	75.5340	87.8610	81.4140	Triclinic
